# Acute Artery of Percheron Infarction: A Case Report Highlighting Diagnostic Challenges and Management

**DOI:** 10.7759/cureus.82072

**Published:** 2025-04-11

**Authors:** Nathan R Barefoot, Andrew R Cunningham, Hayley E Behm, Andrew W Ju, Matthew S Peach

**Affiliations:** 1 Radiation Oncology, Brody School of Medicine at East Carolina University, Greenville, USA

**Keywords:** acute encephalopathy, artery of percheron (aop), artery of percheron infarct, bilateral thalami infarction, ischemic stroke, neuroimaging, thalamic vascular abnormalities

## Abstract

The Artery of Percheron (AOP) is a rare anatomical variant originating from the posterior cerebral artery to supply the bilateral paramedian thalamus and rostral midbrain. AOP infarcts are rare, present with variable symptoms, and are often not detected by conventional neuroimaging, which poses challenges for early diagnosis. This case study presents a 58-year-old male who presented with acute encephalopathy and a Glasgow Coma Scale score of 8. Initial imaging with CT and CTA was negative for an acute intracranial process. MRI revealed bilateral paramedian thalamic and left midbrain ischemia consistent with an AOP infarct. The patient was not a candidate for thrombolytics because his symptoms began nine hours prior to first medical contact, which falls outside of the 4.5-hour therapeutic window for thrombolytics. In addition, his negative CTA precluded his candidacy for thrombectomy. At the time of discharge, the patient’s encephalopathy had improved, but he had residual dysphagia, unintelligible speech, and required assistance with his activities of daily living. This case underscores the diagnostic difficulty of AOP infarcts due to their atypical presentation and the limitations of early imaging modalities. The variability in clinical presentation necessitates a high index of suspicion, as well as the use of advanced imaging techniques, for a timely diagnosis. Very few patients are diagnosed within the therapeutic window for thrombolytics due to initially negative CT and CTAs. Early clinical suspicion should warrant MRI for diagnostic confirmation. Optimal management for patients who fail to meet criteria for thrombolytics, and endovascular intervention is challenging because there are no universally accepted guidelines for the management of AOP infarcts. Early recognition with MRI and tailored management are crucial for optimizing patient outcomes in cases of rare cerebrovascular anomalies.

## Introduction

The thalamus and adjacent midbrain have a complex vascular supply, primarily from branches originating from the posterior cerebral artery (PCA) and posterior communicating artery (PComA). Despite significant overlap, thalamic blood supply is traditionally categorized into four territories: anterior, paramedian, inferolateral, and posterior. The paramedian territory is supplied by paramedian (thalamoperforating) arteries that arise from the P1 segment of the PCA, defined as the proximal PCA segment between the PCA origin and PComA. While the number and territorial distribution of perforating arteries are variable, four defined variants exist. The most common is Type I, which features multiple perforating arteries originating from bilateral P1 segments to supply the bilateral paramedian thalamus and often the rostral midbrain. In rare cases, a single arterial trunk known as the artery of Percheron (AOP) arises from a unilateral P1 segment and gives rise to multiple perforating branches that supply this territory [1-6].

The true prevalence of the AOP is not known. Based on cadaveric studies, it is estimated to be between 4% and 12% [6,7]. Acute ischemia of the AOP can lead to bilateral paramedian infarction with or without midbrain involvement, and, in rare cases, the anterior thalamus [1,8]. AOP infarctions are estimated to occur in 0.1% to 0.6% of all ischemic strokes and account for 4% to 18% of all thalamic strokes [1,9-11].

While the clinical presentation of acute AOP infarction varies, a classic symptomatic triad includes altered mental status, vertical gaze palsy, and memory impairment [1]. Seven characteristic symptom patterns include: mental status disturbances, behavioral amnesic impairment, aphasia/dysarthria, ocular motility disorders, motor deficits, cerebellar signs, and others [3,8]. Non-contrast computed tomography (CT), and CT angiography (CTA) have limited sensitivity for identifying acute AOP ischemia [12]. Due to symptom variability, low incidence rate, and limited neuroimaging sensitivity, acute AOP infarcts are often mistaken for other etiologies of acute encephalopathy. Therefore, diagnosis and treatment of AOP infarctions are commonly delayed and occur outside of the therapeutic window for thrombolytics [5,13-15].

This article was previously presented as a poster at the 26th Annual Neuroscience Symposium at East Carolina Heart Institute on October 31, 2024.

## Case presentation

A 58-year-old male presented to the East Carolina University Medical Center with unresponsiveness and a Glasgow Coma Scale (GCS) score of 8. His past medical history includes a right middle cerebral artery stroke with residual left hemiparesis and dysarthria, focal motor seizures with facial twitching, chronic obstructive pulmonary disease (COPD), coronary artery disease, and chronic tobacco use. Prior to arrival, the patient was found on the floor next to his bed by family, with his last known normal status approximately nine hours earlier. The patient was gently returned to bed by family and was verbally interactive at that time. He became unresponsive to name-calling and gentle shaking two to three hours later, at which point EMS was called. His initial GCS with EMS was 8 (E1V2M5), and he was somnolent but maintained his airway.

In the emergency department (ED), a code stroke was initiated. The patient was minimally responsive, resisting eye-opening and curling up onto the side of the bed with both upper extremities clenched into fists. Deep painful stimuli elicited greater movement on his right side than on his left, though this finding was consistent with the patient's baseline left upper extremity hemiparesis. There was no cyanosis, abnormal jerking of the extremities, muscle stiffness, tongue-biting, urinary or fecal incontinence, or other evidence of a post-ictal episode. The Neurology team’s examination confirmed the findings from the ED team.

**Figure 1:**
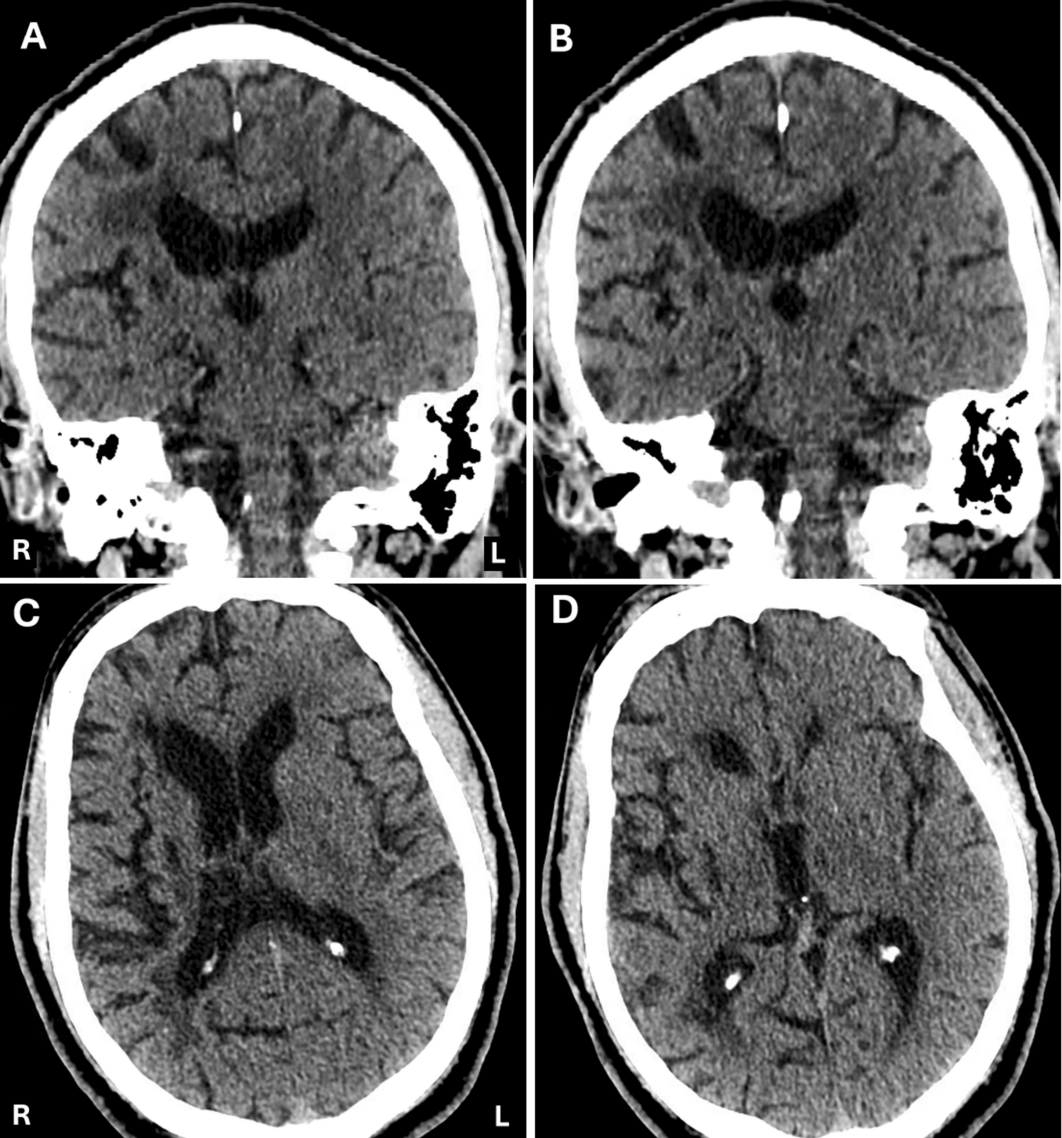
CT Head without contrast performed in the ED No acute intracranial abnormalities were seen in the coronal (A, B) or transverse (C, D) images.

The patient’s imaging and laboratory workup in the ED were as follows. CT Head without contrast showed no acute intracranial abnormality or hemorrhage (Figures 1A-1D). CTA Head and Neck demonstrated no large vessel occlusion or stenosis, as well as an improved appearance of previous mild-to-moderate narrowing of the distal M1 segment. EEG showed no epileptiform discharges or seizures but evidence of moderate diffuse slowing, suggesting diffuse or bilateral cerebral dysfunction. The patient’s carbamazepine level was within the therapeutic range. His creatine phosphokinase (CPK), troponins, lactate, ammonia, blood ethanol, hepatic function panel, and urine drug screen were all negative.

The patient was admitted to the general medicine floor for workup of acute encephalopathy with a broad differential diagnosis, including metabolic, intoxication, stroke, seizure, and factitious disorder. The patient’s family reported that he had a history of factitious behavior. He was started on a loading dose of levetiracetam (2,000 mg IV) and lorazepam (1 mg IV).

**Figure 2:**
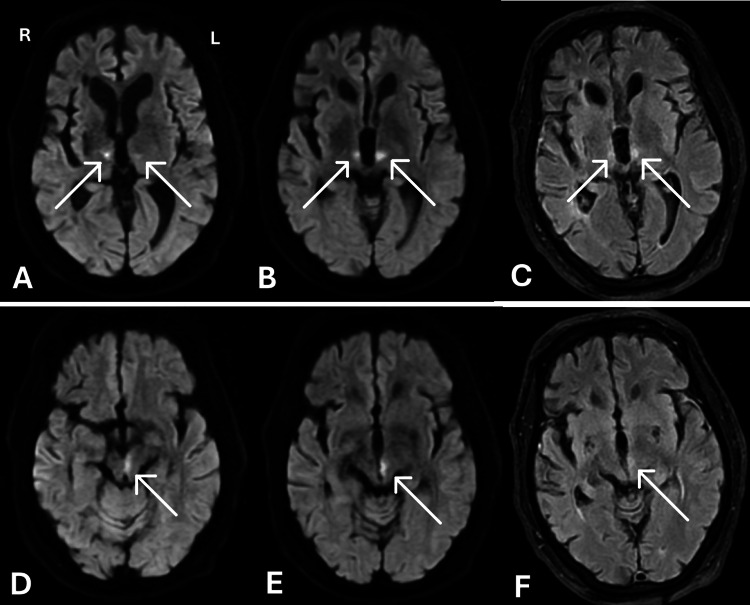
MRI performed on hospital day 1 Axial DWI (A-E) and FLAIR (C, F) images at the level of the thalamus (A-C) and midbrain (D-F). At the level of the thalamus, the arrows point to restricted diffusion involving the bilateral medial thalami, left greater than right, on DWI (A, B) and corresponding FLAIR signal hyperintensity (C). The arrow at the level of the midbrain points to left-sided restricted diffusion extending inferiorly along the left medial midbrain on DWI (D, E) and corresponding FLAIR signal hyperintensity (F).

On hospital day 1, magnetic resonance imaging (MRI) of the brain was performed, which showed an ischemic pattern consistent with an AOP infarct, likely secondary to intracranial small vessel disease (Figures 2A-2F). This interpretation from radiology was not available until hospital day 2. He was started on dual antiplatelet therapy with ticagrelor 90 mg twice daily, scheduled for 90 days, and was to continue lifelong daily aspirin 81 mg. An amantadine taper was started to help with mentation and was completed prior to discharge. His carbamazepine dosage was changed from 400 mg PO twice daily to 250 mg PO three times daily per Neurology's recommendations. No breakthrough seizures occurred during his admission. A swallow study showed no dysphagia, and enteral feeding was discontinued.

On hospital day 2, the patient showed improvement: he opened his eyes in response to name-calling, attempted to track with his eyes, and responded verbally, though his speech was unintelligible. His face was symmetrical. Cranial nerves II-XII were intact bilaterally. He followed verbal commands, such as raising his arm to give a “high five.” There were no lower extremity motor deficits. He continued to have left upper extremity weakness consistent with his baseline.

On hospital day 3, the patient developed new right-sided weakness, with loss of the right nasolabial fold and left eye ptosis. Repeat CTA demonstrated no significant change from the prior CTA. CT venography showed no evidence of dural venous thrombosis, and echocardiography showed no evidence of intracardiac shunt. Although the etiology of these new-onset symptoms was unclear, the patient had no further neurological decline. These symptoms gradually improved throughout the patient’s stay but were still evident at discharge. The patient was discharged to a skilled nursing facility on hospital day 15. While his encephalopathy had improved, he had residual dysphagia and unintelligible speech, and he required assistance with his activities of daily living.

## Discussion

**Figure 3:**
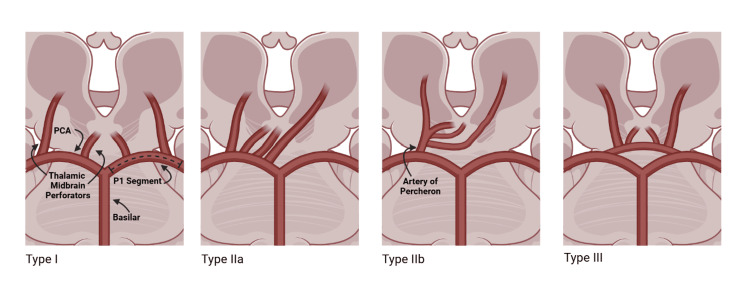
Variants of paramedian thalamic blood supply Type I: normal anatomy with multiple thalamic and midbrain perforating arteries originating from bilateral P1 segments. Type IIa: multiple perforating arteries originate from a unilateral P1 segment. Type IIb: single Artery of Percheron originates from unilateral P1 segment. Type III: known as the arcade variant, in which an arterial arc connects both P1 segments and gives off multiple perforating branches. Image Credits: H. Behm (author) [18]

At least two of the three hallmark symptoms - altered mental status, vertical gaze palsy, and memory impairment - are present in most AOP stroke patients [8,14-16]. Mental status disturbance was the only hallmark symptom in our patient, but a thorough neurological exam was limited due to poor cooperation and unintelligible speech. Mental status disturbance is the most common symptom and can range from drowsiness to coma. Ocular motility disorders may include gaze and cranial nerve palsies, ophthalmoplegia, and pupillary defects. Aphasia or dysarthria is also common [8,14,16]. Initial GCS and National Institutes of Health Stroke Scale (NIHSS) scores range from 4 to 15 and from 1 to 40, respectively [15]. A literature review of cases from 1985 to 2006 found vertical gaze palsy (65%), memory impairment (58%), and coma (42%) to be the most common presenting symptoms [17]. However, to the authors’ knowledge, there are no larger studies or recent literature reviews that assess symptom frequency. The varying territorial distribution of thalamic blood supply from an AOP may contribute to the range of presenting symptoms (Figure 3).

Non-contrast CT and CTA are commonly used as first-line imaging for stroke diagnosis due to their availability, high sensitivity for hemorrhage, and cost-effectiveness. However, these modalities have limited sensitivity for identifying acute AOP ischemia, with non-contrast CT sensitivity estimated to range between 23% and 55% [8,12,15,19-21]. Conventional angiography does not routinely image an AOP; therefore, it may not be visualized if occluded [3,22]. MRI, particularly fluid-attenuated inversion recovery (FLAIR) and diffusion-weighted imaging (DWI), is the preferred method for detecting acute AOP infarcts [13]. However, there are two reports where the initial MRI was negative, suggesting that a normal initial MRI does not definitely exclude the diagnosis [23,24]. One review recommends repeat imaging within 48 hours if clinical suspicion is high and the initial MRI is negative [25].

Lazzaro et al. assessed imaging from 37 patients and described four distinct patterns and frequencies of infarction: bilateral paramedian thalamus with rostral midbrain (43%), bilateral paramedian thalamus without midbrain (38%), bilateral paramedian and anterior thalamus with midbrain (14%), and bilateral paramedian and anterior thalamus without midbrain (5%) [1]. Smaller case series demonstrate a similar frequency of infarct patterns, with bilateral paramedian thalamus with or without midbrain involvement accounting for over 80% of presentations [8,15]. Sixty-seven percent of cases with midbrain involvement present with a pathognomonic “V sign,” a high-intensity signal observed on FLAIR and DWI [1].

For ischemic strokes, the American Heart Association and American Stroke Association recommend administering IV tissue plasminogen activator (tPA) within 3 to 4.5 hours of symptom onset [26]. However, as demonstrated in our patient, AOP infarcts are rarely diagnosed within this critical window. The average time from symptom onset to radiographic diagnosis is 1.9 days (median 1.1 days) with a range from 3.5 hours to 4.5 days [14]. Our literature review yielded five patients who were treated with tPA, two of whom were treated six hours after symptom onset. All patients showed significant improvement in functional status post-thrombolysis. Of these, three patients recovered completely, one had residual vertical gaze paresis, and one remained severely cognitively impaired [14,22,27-29]. Notably, none of these patients experienced hemorrhagic transformation following tPA.

In rare cases, unilateral occlusion of the proximal P1 segment in patients with an AOP manifests as an AOP infarction. The recommended management for proximal PCA occlusions is IV tPA within 4.5 hours and mechanical thrombectomy within six hours. Patients who are not eligible for tPA may still benefit from mechanical thrombectomy [30]. Our literature review also found five patients who underwent endovascular intervention. One patient, who presented with coma, tetraparesis, and a GCS of 8, was treated with both tPA and mechanical thrombectomy. His symptoms rapidly resolved, and he was discharged home with a Modified Rankin Score (mRS) of 0 [29]. Of the four patients treated with mechanical thrombectomy alone, three had mRS scores at discharge of 1, 2, and 3, respectively, while one patient was transferred to hospice [29,31,32].

The treatment protocol for patients ineligible for thrombolytics and endovascular intervention is poorly defined. One study proposes a treatment algorithm for emergent and non-emergent AOP infarcts based on a review of six cases. It recommends treating emergent cases outside of the tPA therapeutic window with IV heparin followed by long-term anticoagulation. For non-emergent cases not involving the midbrain, long-term anticoagulation and rehabilitation are advised, while cases involving the midbrain should receive IV heparin [25]. However, the evidence supporting these interventions is limited, and the study fails to clearly define what constitutes emergent versus non-emergent AOP infarcts. Ultimately, treatment should focus on addressing the underlying etiology. For example, one case of an AOP infarct from a cardioembolic source due to non-valvular atrial fibrillation was treated with oral anticoagulation [33].

Stamm et al. assessed the hospital course of 12 patients with AOP infarcts, reporting an average length of stay of 8.3 days (range: three to 14 days). While in the hospital, eight patients were in the ICU and had an average ICU stay of 2.9 days (range: one night to eight days). Four of these patients required intubation for an average intubation time of 3.7 days. The four non-ICU patients were treated by the general medicine team. Regarding disposition, four patients were discharged home, three were transferred to inpatient rehabilitation, and five were discharged to a skilled nursing facility [14].

While AOP infarcts are typically not fatal, patients often suffer long-term behavioral and cognitive deficits. Midbrain involvement is associated with worse outcomes, notably in executive processing, memory, and linguistic abilities [16]. One study found that 67% of infarcts without midbrain involvement had a favorable outcome compared to 25% of those with midbrain involvement; a favorable outcome was defined as an mRS ≤ 3 and independence in activities of daily living [8].

This study’s findings are limited by potential selection bias due to reliance on retrospective case series and literature reviews for comparison. The case study’s single-patient focus restricts its broader generalizability. There is a need for a comprehensive review and established recommendations for this rare presentation by experts from high-volume stroke centers and neurologists.

## Conclusions

Occlusion of the AOP is a rare form of ischemic stroke with a myriad of presenting symptoms and variable imaging findings, often resulting in delayed diagnosis. This case underscores the importance of considering AOP infarction in patients - particularly elderly individuals - with unexplained altered mental status, vertical gaze palsy, and/or memory impairment. Clinicians should maintain a high index of suspicion for AOP infarction when these symptoms present with a negative CT and therefore should have a low threshold for emergent MRI with FLAIR and DWI. Both the current case and a review of similar cases in the literature suggest that early recognition and timely intervention with thrombolytics and/or mechanical thrombectomy may improve clinical outcomes. Moreover, there may be potential benefit from late intervention and therapy, even outside the standard therapeutic window. For clinicians in emergency or neurology settings, early recognition of AOP infarction and a proactive approach to imaging and treatment are key to optimizing patient recovery. Further research is needed to develop comprehensive, evidence-based guidelines for managing AOP infarctions, particularly in patients who do not meet the standard criteria for thrombolytic therapy.
